# Cognitive performance of long-term institutionalized elderly patients with schizophrenia: A case control study

**DOI:** 10.1590/S1980-57642011DN05020007

**Published:** 2011

**Authors:** Alexandre Paim Diaz, Monica Zavaloni Scalco, Marcelo Libório Schwarzbold, Douglas Affonso Formolo, Alberto Stoppe Júnior

**Affiliations:** 1 Núcleo de Pesquisas em Neurologia Experimental e Clínica (NUPNEC), Departamento de Clínica Médica, Universidade Federal de Santa Catarina, Florianópolis SC, Brazil and Instituto de Psiquiatria de Santa Catarina, São José SC, Brazil; 2 Department of Psychiatry, University of Toronto and Whitby Mental Health Centre, Seniors Program, Canada; 3 Núcleo de Pesquisas em Neurologia Experimental e Clínica (NUPNEC), Departamento de Clínica Médica, Universidade Federal de Santa Catarina, Florianópolis SC, Brazil; 4 Núcleo de Pesquisas em Neurologia Experimental e Clínica (NUPNEC), Departamento de Clínica Médica, Universidade Federal de Santa Catarina, Florianópolis SC, Brazil; 5 Ambulatório de Neuropsiquiatria Geriátrica, Florianópolis SC, Brazil

**Keywords:** elderly, cognition, institutionalization, schizophrenia

## Abstract

Cognitive impairment is inherent to the ageing process. Several studies suggest that patients with late-life schizophrenia have more marked cognitive impairment.

**Objective:**

The aim of this study was to compare the cognitive performance of elderly institutionalized patients with schizophrenia and institutionalized elderly control patients without neurological or psychiatric diseases, matched for age, educational level and institutionalization time.

**Methods:**

The Cambridge Examination for Mental Disorders of the Elderly (CAMCOG) was used to test 10 institutionalized elderly patients with schizophrenia. Results were compared with those of 10 institutionalized control patients with history of Hansen’s disease.

**Results:**

Patients with schizophrenia showed a worse performance in terms of total CAMCOG score and on its subtests of orientation, language, abstraction, and memory (p≤0.05). Patients with schizophrenia also disclosed a non-significant trend toward lower scores on the MMSE and on calculus.

**Conclusion:**

Findings demonstrated that schizophrenia was associated to worse cognitive impairment in long-term institutionalized elderly patients compared with institutionalized patients without neurological or psychiatric diseases.

## Abstract (pt)

Prejuízo cognitivo é inerente ao processo de senescência. Estudos tem sugerido que pacientes idosos com esquizofrenia apresentam esse prejuízo de maneira mais acentuada.

**Objetivo:**

O objetivo deste estudo foi comparar o desempenho cognitivo de pacientes idosos com esquizofrenia, institucionalizados, com indivíduos idosos, institucionalizados, sem doenças neurológicas ou psiquiátricas, pareados pela idade, escolaridade e tempo de institucionalização.

**Métodos:**

“Cambridge Examination for Mental Disorders of the Elderly” (CAMCOG) foi aplicado em 10 pacientes, institucionalizados, com esquizofrenia, cujo desempenho cognitivo foi comparado ao de 10 indivíduos, institucionalizados, com história de doença de Hansen.

**Resultados:**

Pacientes com esquizofrenia apresentaram um pior desempenho na pontuação total do CAMCOG e em seus subitens orientação, linguagem, abstração e memória (p≤0.05). Pacientes com esquizofrenia também apresentaram uma tendência, não significativa, para menor pontuação no MEEM e cálculo.

**Conclusão:**

Nossos achados demonstram que a esquizofrenia está associada a piora do comprometimento cognitivo em pacientes idosos com institucionalização de longa permanência comparados a pacientes institucionalizados sem doenças neurológicas ou psiquiátricas.

Cognitive impairment is inherent to the ageing process and may be worse in elderly patients with Schizophrenia.^1,2^ Cross-sectional reports suggest that patients with late-life schizophrenia have more marked impairment of executive function, visuospatial ability, verbal fluency, memory, attention, and working memory.^3^

Cognitive impairment is an important factor related to the outcome in Schizophrenia^4^ and neurocognitive deficits appear to contribute independently to poorer quality of life^5^ and also interfere with social function^6,7^ in these patients.

Some studies have evaluated cognition in institutionalized elderly patients. However, we found no studies based on a comparison group comprising elderly institutionalized individuals without neurological or psychiatric diseases.

The present study analyzed the cognitive performance of institutionalized elderly individuals with schizophrenia on the Cambridge Cognitive Test (CAMCOG)^8^ and compared results against elderly controls without any history of psychotic disorder. Our hypothesis was that elderly patients with Schizophrenia have a worse cognitive performance compared to non-psychotic patients when controlled for age, gender, educational level and living status.

## Methods

### Participants and setting

The group of patient comprised elderly individuals with schizophrenia who were institutionalized at the Psychiatry Institute of Santa Catarina, in São José, SC, Brazil. The exclusion criteria were: current Major Depression Disorder, history of substance-related disorders, previous diagnosis of dementia, current *delirium*, history of epilepsy or cerebral vascular accident, hearing loss, visual or speech deficit, mental retardation, and those presenting a non-stabilized comorbid non-psychiatric medical condition.

Schizophrenia diagnosis was confirmed through a clinical interview, according to the diagnosis criteria of the Diagnostic and Statistical Manual of Mental Disorders (DSM-IV-TR),^9^ and to the patient’s records. All patients received typical antipsychotics and, based on the date of the first registration at a hospital, the average time suffering from the disorder was 37.8 years. Ten out of the 51 institutionalized patients were eligible for the study and agreed to participate. The control group comprised elderly individuals with long-term institutionalization at the Hospital Santa Tereza in São Pedro de Alcântara, SC, Brazil. These individuals had undergone treatment for Hansen disease and were institutionalized at this hospital due to socio-economic reasons or because of the disease stigma. The same criteria for exclusion of the patient group were applied to the controls, with the added condition that controls must have no history of psychotic disorders. These controls were not in use of medications which could impair their cognitive performance. Of the 22 patients over 60 years old, 10 met the inclusion criteria. Among the illiterate patients evaluated, 3 were in the patient group and 1 in the control group. Both institutions were geographically close to one another (approximately 7 km between them) and patients and controls disclosed similar socio-cultural influences.

### Main outcome measures

A cognitive evaluation was carried out using the CAMCOG scale, the cognitive part of The Cambridge Examination for Mental Disorders of the Elderly (CAMDEX).^8^ Item 175 (praxia: sense of touch) was excluded due to the peripheral neurological sequelae presented by the controls. The CAMCOG also includes items from the Mini-Mental State Examination (MMSE).^10^

The CAMCOG was applied by a medical doctor specialized in psychiatry, according to the directions given in the translated version adapted to Portuguese.^11^ Evaluations were carried out during 2006. The groups were compared regarding age, institutionalization time, educational level, total score for the MMSE (ranging from 0 to 30), total score for the CAMCOG (from 0 to 104), and its sub-items: orientation (0 to 10), language (0 to 29), memory (0 to 27), attention (0 to 7), praxia (0 to 12), calculation (0 to 2), abstraction (0 to 8) and perception (0 to 9), where higher scores indicate better cognitive functioning.

The continuous variables were analyzed by Student’s “t” test. Because this first analysis showed a trend toward lower age in the control group, this imbalance was corrected by linear multiple logistic regression. In this analysis, the dependent variables were the lower cognitive scores initially associated to the schizophrenic group, with a “p” level of less than 0.20. After the linear multiple logistic regressions, the association between the cognitive performance test and schizophrenia were considered statistically significant for “p” levels ≤0.05. Given the small sample of both groups studied it was decided not to adjust the “p” level significance for multiple comparisons in order to avoid a type II error. This study was approved by the ethics committees of the Psychiatry Institute of Santa Catarina and informed consent was obtained from all patients or their legal representatives.

## Results

The distribution of clinical-demographic variables is shown in Table 1. There was a non-significant trend (p=0.09) toward older age among the controls. No statistical differences between patients and controls were found according to gender, educational level and institutionalization time. The univariate analysis revealed that patients with schizophrenia disclosed lower scores on the MMSE, total CAMCOG and on its sub-items orientation, language and abstraction (Table 1).

**Table 1:** Distribution of demographic and cognitive variables according to psychiatric diagnosis.

Variables	All cases	Schizophrenics (n=20)	Controls (n=10)	p-level (n=10)
Gender				
Male	12	6	6	
Female	8	4	4	1.0
Age	69.7 (8.4)	66.6 (7.4)	72.9 (8.6)	0.09
Educational level (ys)	3.4 (3.1)	3.6 (3.5)	3.1 (2.7)	0.75
Institutionalization time (ys)	21 (17.1)	16.8 (8.6)	24.8 (7.1)	0.31
MMSE	22 (4.3)	20.1 (4.6)	23.8 (3.2)	0.05
CAMCOG	64.9 (14.2)	57.6 (14.6)	72.2 (9.5)	0.02
Orientation	8.2 (1.6)	7.5 (1.8)	9.0 (0.7)	0.03
Language	22.6 (4.4)	20.2 (4.7)	25.1 (2.4)	0.01
Abstraction	1.8 (2.0)	0.4 (0.7)	3.2 (1.8)	0.001
Memory	14.7 (5.0)	12.9 (4.6)	16.5 (4.8)	0.11
Attention	3.3 (2.7)	2.6 (3.1)	4.1(2.2)	0.28
Praxis	7.9 (1.9)	7.8 (1.6)	8.1 (2.3)	0.74
Calculation	1.4 ( 0.6)	1.2 (0.6)	1.6 (0.5)	0.14
Perception	4.8 (1.8)	5.0 (2.4)	4.6 (1.3)	0.26

Table 2 shows the final models of multiple logistic regressions that best explained the association between schizophrenia and lower score on the cognitive tests. After correcting for age imbalance between patients and controls, the analysis showed a lower patient performance on the CAMCOG, orientation, language, abstraction and memory (p≤0.05). A non-significant trend toward lower scores on the MMSE (p=0.09) and calculation (p=0.10) was evident.

**Table 2:** Final model of multiple logistic regressions best explaining association between lower score on cognitive tests and schizophrenia.

Variables	Mean square	F value	p-level
MMSE			
Age	1.3	0.08	0.78
Group	51.9	3.07	0.09
CAMCOG			
Age	72.99	0.46	0.50
Group	1117.57	7.09	0.02
Orientation			
Age	0.25	0.12	0.73
Group	8.45	4.19	0.05
Language			
Age	0.10	0.007	0.93
Group	99.98	6.89	0.02
Abstraction			
Age	0.004	0.002	0.96
Group	33.74	16.87	0.001
Memory			
Age	44.56	2.10	0.17
Group	99.85	4.70	0.04
Calculation			
Age	0.18	0.54	0.47
Group	0.98	2.87	0.10

## Discussion

These findings are consistent with other results in the literature showing generalized cognitive impairment in schizophrenia. Several studies have demonstrated that domains such as executive/abstracting functioning, verbal and non-verbal fluency, verbal and visual-spatial working memory, attention, besides other cognitive functions, can be impaired in patients with Schizophrenia.^2,12,13^

In the present investigation, both study groups had low scores on the MMSE and the CAMCOG. Based on a Brazilian study^14^ that proposed normal scores on the MMSE according to educational level, 80% of our patients scored below normal. This finding is in line with results of another Brazilian study^15^ which evaluated elderly patients with schizophrenia in a 1-year follow-up. In the cited study, the baseline MMSE measurement for the sample was lower than that recommended as normal for the Brazilian elderly population.

Both groups share characteristics that could explain their low cognitive performance. Living situation and stimuli individuals are exposed to have been investigated as potential influences on cognitive functions. Our patients and controls were institutionalized in environments with fewer stimuli (especially patients) compared to individuals living in the community, a factor which could have influenced the results. Studies in mice^16^ placed in rich and non-rich stimuli environments showed that environmental stimuli had a positive influence on attention tests and hippocampus-mediated behavior. In addition, the brain levels of several structures and synaptic proteins involved in neurite outgrowth, cell survival and sinaptogenesis, which are associated to cognitive functions, were affected by rich-environmental factors.^17^

The fact that our patients were institutionalized may indicate they had more serious conditions, where this could contribute to their low cognitive performance thus explaining their inability to live independently.^18^ However, it was clear that one of the main reasons for the patients being institutionalized was related to social issues, and not necessarily to worse psychotic disease.

Educational level (years of schooling) may also be associated to lower cognitive performance. In a Brazilian study^19^ using the MMSE, the authors evaluated cognition in 73 elderly schizophrenic institutionalized patients and found a significant association between years of schooling and MMSE performance. Findings in this study showed that even a very low level of formal education was associated to better cognitive performance in the sample assessed.

Other factors can also contribute to cognitive deficits in patients with chronic schizophrenia. In a review, Sacchetti et al. (2010) pointed out that antipsychotic medications could increase the risk of cardiovascular events in patients under psychiatric treatment.^20^ Clinical comorbidity is known to be frequent among schizophrenic patients, such as type 2 diabetes and high blood pressure.^21,22^ This population also has difficulty achieving good control of blood pressure and blood sugars,^23,24^ largely because of lack of compliance with recommended treatments, but also due to increased cardiovascular risk and metabolic syndrome associated with the use of antipsychotic medications. Therefore, these clinical comorbidities, especially when not well-controlled, can also lead to cognitive deficits in patients with schizophrenia.

The present study had several limitations, the most significant of which was the small sample size. However, advantages of the study include having compared groups that were similar according to gender and education, and adequately controlled for age imbalance. Also, all the individuals were institutionalized, without significant differences regarding the institutionalization time, and had similar cultural socio-cultural characteristics. Finally, the patients’ pharmacological treatments, which included typical antipsychotic medication as well as anticholinergics, may have also interfered in subjects’ cognitive performance.^25,26^

This is the first report investigating cognitive performance in institutionalized elderly schizophrenic patients compared to institutionalized mentally healthy elders. Results revealed significantly worse performance among the patient group. The association between institutionalization and cognitive performance in elderly with and without psychotic symptoms warrants further investigation.
